# Microsporidial Stromal Keratitis: Successful Treatment with Topical Voriconazole and Oral Itraconazole

**DOI:** 10.7759/cureus.934

**Published:** 2016-12-20

**Authors:** Mircea Coca, James Kim, Sudhir Shenoy, Patricia Chévez-Barrios, Manuj Kapur

**Affiliations:** 1 Chicagoland Retinal Consultants; 2 Ophthalmology, UTMB; 3 Ophthalmology, Froedtert Eye Institute, Medical College of Wisconsin, Milwaukee, WI; 4 Texas Tech University Health Sciences Center; 5 Department of Pathology and Genomic Medicine, Department of Ophthalmology, Houston Methodist Hospital, Houston, TX; 6 Coastal Eye Associates

**Keywords:** microsporidium, stromal keratitis, medical treatment, voriconazole, itraconazole, cornea

## Abstract

We report a case of microsporidial stromal keratitis successfully treated with topical voriconazole and oral itraconazole. A 30-year-old Hispanic male construction worker who wears contacts lenses presented with left eye erythematous, epiphora, and mild pain increasing over few days after failing previous antibiotics treatment. His best corrected visual acuity in the left eye was count fingers at three feet, and the slit lamp examination showed 3+ conjunctival injection, a circular central corneal ulcer 3.2 mm in diameter, stromal thinning, and an anterior chamber with white cells, flair, and 0.1 mm hypopyon. A cornea punch biopsy identified microsporidial organisms with some features suggestive of Vittaforma corneae. After treatment with topical voriconazole and oral itraconazole for eight weeks, the patient had complete resolution with no recurrence for over 12 months of follow-up. To our knowledge, this is the first reported case of successful treatment of microsporidial stromal keratitis with antifungals.

## Introduction

Microsporidial keratitis is a rare condition with poor medical response and no treatment guidelines. Almost all reported cases of microsporidial stromal keratitis failed medical therapy and required penetrating keratoplasty. We report a case of stromal keratitis caused by microsporidia that was successfully treated by topical voriconazole and oral itraconazole combination therapy. To our knowledge, this is the first case of microsporidial stromal keratitis that has been successfully treated with topical voriconazole and oral itraconazole combination therapy and the third reported case of successful medical therapy of microsporidial stromal keratitis leading to complete resolution. Informed consent was obtained from the patient for this study.

## Case presentation

A 30-year-old Hispanic male construction worker who wears contacts lenses reported feeling something fall into his left eye at work. He reported cleaning the contact lens with contact lens cleaning solution before reinserting the contact into his eye and returning to work. The next morning his left eye was erythematous, lacrimating, and mildly painful. He was seen by an optometrist and prescribed neomycin, polymyxin B and dexamethasone ophthalmic ointment, and moxifloxacin ophthalmic solution with no resolution of symptoms. Three days later, the patient presented to our institution and his best corrected visual acuity (BCVA) in the left eye (OS) was count fingers at three feet. A slit lamp examination showed 3+ injection of the conjunctiva, a circular central corneal ulcer 3.2 mm in diameter with peripheral haze, stromal thinning, and an anterior chamber with white cells, flair, and 0.1 mm hypopyon. The corneal epithelium was scraped for culture and the patient was initially started on fortified vancomycin (25 mg/mL) ophthalmic solution and fortified tobramycin (15 mg/mL) ophthalmic solution one drop every one hour after receiving one time subconjunctival vancomycin (25 mg) and tobramycin (25 mg) injections.

**Figure 1:**
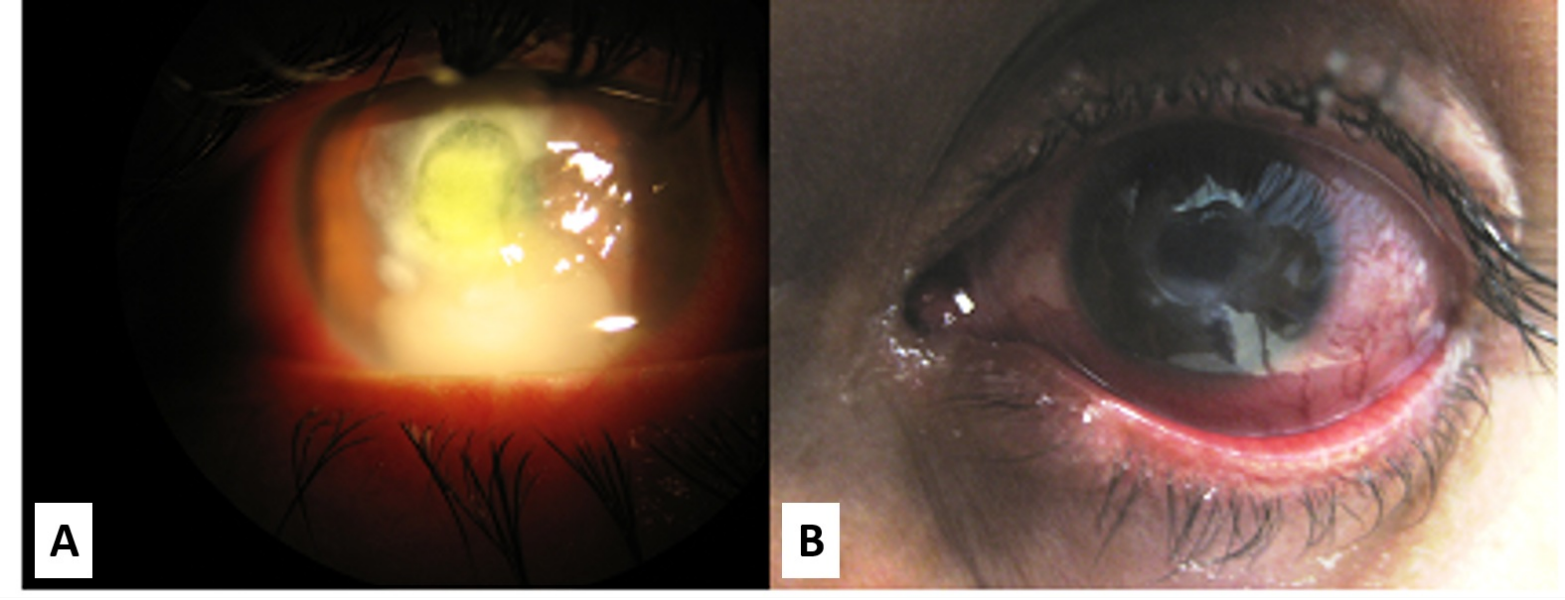
Slit lamp images of the cornea early on and after the resolution of stromal keratitis A) Slit lamp image at day 21 of infection demonstrating corneal ulceration and hypopyon. B) Image at eight weeks after start of topical voriconazole and oral itraconazole therapy showing complete microbiological clearance of stromal keratitis.

**Figure 2:**
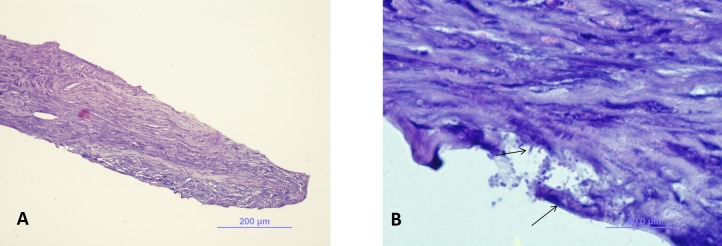
Light microscopy of corneal biopsy A) A low power view of the biopsy shows that the deep stroma contains groups of microsporidial oval spores in the stromal spaces. (Hematoxylin-Eosin, original magnification 10X.) B) Higher magnification shows the oval spores of microsporidia in the spaces of the deep stroma. (Hematoxylin-Eosin, 40X original magnification).

**Figure 3:**
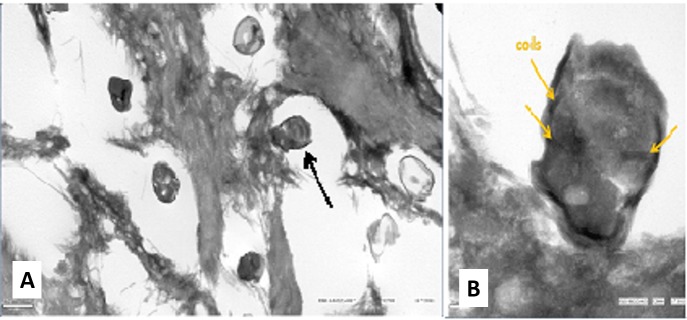
Transmission electron microscopy (TEM) of the corneal biopsy A) Transmission electron microscopy (TEM) low power view shows multiple spores measuring about 1.5-2 microns in length. B) TEM high power view shows coils (dark string-like structures) classic for microsporidia and features suggestive of two abutting nuclei*.*

During the following days, the ulcer increased in size and the hypopyon increased to 3 mm (Figure 1A). Potassium hydroxyde preparation and gram stain were negative for microorganisms. Corneal cultures showed growth of *Streptococcus pneumoniae* susceptible to cefazolin. Subsequently, subconjunctival cefazolin (100 mg) was administered and the patient was started on cefazolin ophthalmic solution. Despite 10 days of topical cefazolin therapy, the infiltrate continued to enlarge. Multiple punch biopsies were obtained of which only one containing deeper stroma demonstrated microsporidial spores on routine stains (Figures 2A, 2B). Electron microscopy obtained to confirm diagnosis from the slide of the biopsy identified microsporial organisms with some features suggestive of *Vittaforma corneae* but the fixation artifact precluded definite classification (Figures 3A, 3B). The patient was started on voriconazole ophthalmic solution (one percent) one drop every one hour and oral itraconazole 200 mg twice daily. With continued therapy for three weeks, the patient showed marked improvement. By the eighth week of treatment, there was complete resolution of microsporidial stromal keratitis (Figure 1B). The patient has not had a recurrence over 48 months of follow-up.

## Discussion

Microsporidia are small (1-40 μm), spore-forming, obligate intracellular fungal parasites of the phylum Microspora with over 1200 species, of which 14 infect humans [1]. Microsporidia can infect various systems including intestinal, respiratory, ocular, urinary, muscular, and central nervous system [2]. The microsporidial genera of *Encephalitozoon *and *Nosema*  (renamed *Vittaforma*) are most commonly known to cause ocular infections in humans [2]. The risk factors for ocular microsporidial infections include systemic immunosuppression, contact lens wear, topical corticosteroid use, ocular trauma with dust or insect bite, chronic sinusitis, contact with domestic animals, and exposure to contaminated food, water, or soil [1-2]. The *Encephalitozoon* genus is associated with diffuse punctate epithelial keratoconjunctivitis usually affecting HIV-infected individuals. Superficial keratoconjunctivitis caused by *Encephalitozoon* has been shown to be successfully treated with medical therapy including antibiotics and antifungals [2]. The genus *Vittaforma* is associated with corneal stromal keratitis affecting immunocompetent individuals usually requiring penetrating keratoplasty (PKP) for resolution [2-4]. To our knowledge, there are three cases of stromal keratitis that have been successfully treated with medical therapy in the same institution: one with systemic albendazole and topical chlorhexidine [5], and two with a combination of topical antiparasitic agents such as polyhexamethylene biguanide (PHMB) and chlorhexidine instillations with or without oral albendazole or itraconazole [6].

Microsporidial stromal keratitis has a poor prognosis and a high rate of medical therapy failure requiring PKP in most reported cases in literature. It is often mistaken as bacterial, viral, or fungal keratitis, and targeted treatment for microsporidia is often delayed. Microsporidial organisms do not grow in cultures and the diagnosis is usually confirmed by cytologic or histopathologic observation of the organisms. Microsporidia is a very small organism and unless the pathologist has the organism in the differential diagnosis it may pass undiagnosed. Past medical treatments for microsporidial stromal keratitis included topical chloramphenicol, fumagillin, oral albendazole or itraconazole, thiabendazole, propamidine isethionate, chlorhexidine, metronidazole, polyhexamethylene biguanide, and benzimidazole [2]. Sangit, Murthy, Garg reported a case of stromal keratitis in a 14-year-old girl who was successfully treated with topical chlorhexidine and systemic albendazole for 12 weeks [5]. Garg also reported two more cases of stromal keratitis treated with a combination of systemic albendazole and another medication not specified in the report [6]. Except the aforementioned three cases, all medical therapies failed to control stromal microsporidial keratitis and required PKP.

Khandelwal, et al. reported two cases of microsporidial epithelial keratitis successfully treated with topical voriconazole monotherapy [7]. Voriconazole has a wide antifungal spectrum and excellent ocular penetration that is ideal for stromal keratitis. Also microsporidia has recently been reclassified from parasite to a fungus, further supporting the role of voriconazole and other antifungals as possible therapies for ocular microsporidial infections [7].

As demonstrated in our case report, diagnosing microsporidial stromal keratitis is challenging. Given the initial presentation of the patient with contact lens use and possible ocular trauma with a foreign body, bacterial, fungal, acanthamoeba or viral keratitis was initially suspected based on the most common causes of keratitis. However, with failed antibiotic therapy and negative cultures, suspicion for microsporidial infection grew higher on the differential diagnosis. Multiple punch biopsies were needed to confirm the diagnosis before targeted treatment could be initiated as the organisms are often located in deeper stroma. It is also important that the pathologist is aware of the entity to find the organisms that are often not associated with the inflammatory infiltrate or necrosis. After undergoing topical voriconazole and oral itraconazole treatment for eight weeks, the patient had complete resolution with no recurrence for over 12 months now.

## Conclusions

To the best of our knowledge this is the first case of stromal microsporidial keratitis to be successfully treated with topical voriconazole and oral itraconazole combination therapy resulting in complete resolution. If diagnosed early and treated appropriately with medical therapy, patients with microspordial stromal keratitis might not require PKP as an end result. Further studies regarding the use of topical voriconazole with oral itraconazole combination therapy for treatment of microsporidial-related stromal keratitis need to be investigated to determine the efficacy of treatment and to monitor for recurrence of infection. The role of multiple punch biopsies and corneal scrapings before initiation of therapy also needs to be studied to determine if penetration of the drug therapy into the stroma plays a role in the resolution of the infection.
